# Supplementary material for: Medicinal Hypervalent Tellurium Prodrugs Bearing Different Ligands: A Comparative Study of the Chemical Profiles of AS101 and Its Halido Replaced Analogues
Source: Int J Mol Sci. 2022 Jul 6;23(14):7505. doi: 10.3390/ijms23147505 (PMC9317073; doi:10.3390/ijms23147505)
Supplement: Supplementary file 1 [file ijms-23-07505-s001.zip › ijms-1793848-supplementary.pdf]

Medicinal Hypervalent Tellurium Prodrugs Bearing Different Ligands: A Comparative Study of the Chemical Profiles of AS101 and Its Halido Replaced Analogues

Lorenzo Chiaverini [1,†], Damiano Cirri [2,†], Iogann Tolbatov [3,*], Francesca Corsi [1], Ilaria Piano [1], Alessandro Marrone [4], Alessandro Pratesi [2], Tiziano Marzo [1,*] and Diego La Mendola [1]

[1] Department of Pharmacy, University of Pisa, Via Bonanno Pisano, 6, 56126 Pisa, Italy; lorenzo.chiaverini@farm.unipi.it (L.C.); francesca.corsi@phd.unipi.it (F.C.); ilaria.piano@unipi.it (I.P.); diego.lamendola@unipi.it (D.L.M.)
[2] Department of Chemistry and Industrial Chemistry, University of Pisa, Via G. Moruzzi, 13, 56124 Pisa, Italy; damiano.cirri@dcci.unipi.it (D.C.); alessandro.pratesi@unipi.it (A.P.)
[3] Institute of Chemical Research of Catalonia (ICIQ), The Barcelona Institute of Science and Technology, 43007 Tarragona, Spain
[4] Dipartimento di Farmacia, Università degli Studi "G. D'Annunzio" Chieti-Pescara, Via dei Vestini, 66100 Chieti, Italy; amarrone@unich.it
* Correspondence: tolbatov.i@gmail.com (I.T.); tiziano.marzo@unipi.it (T.M.)
† These authors contributed equally to this work.

Contents

NMR spectra 2
IR spectra 7
Isolation of the oxidized species 10
Computational studies 11
Biological studies 12

NMR spectra

General statement: NMR spectra have been recorded carefully using anhydrous DMSO. However, the residual presence of water partially affects the analysis and a partial conversion is detectable.

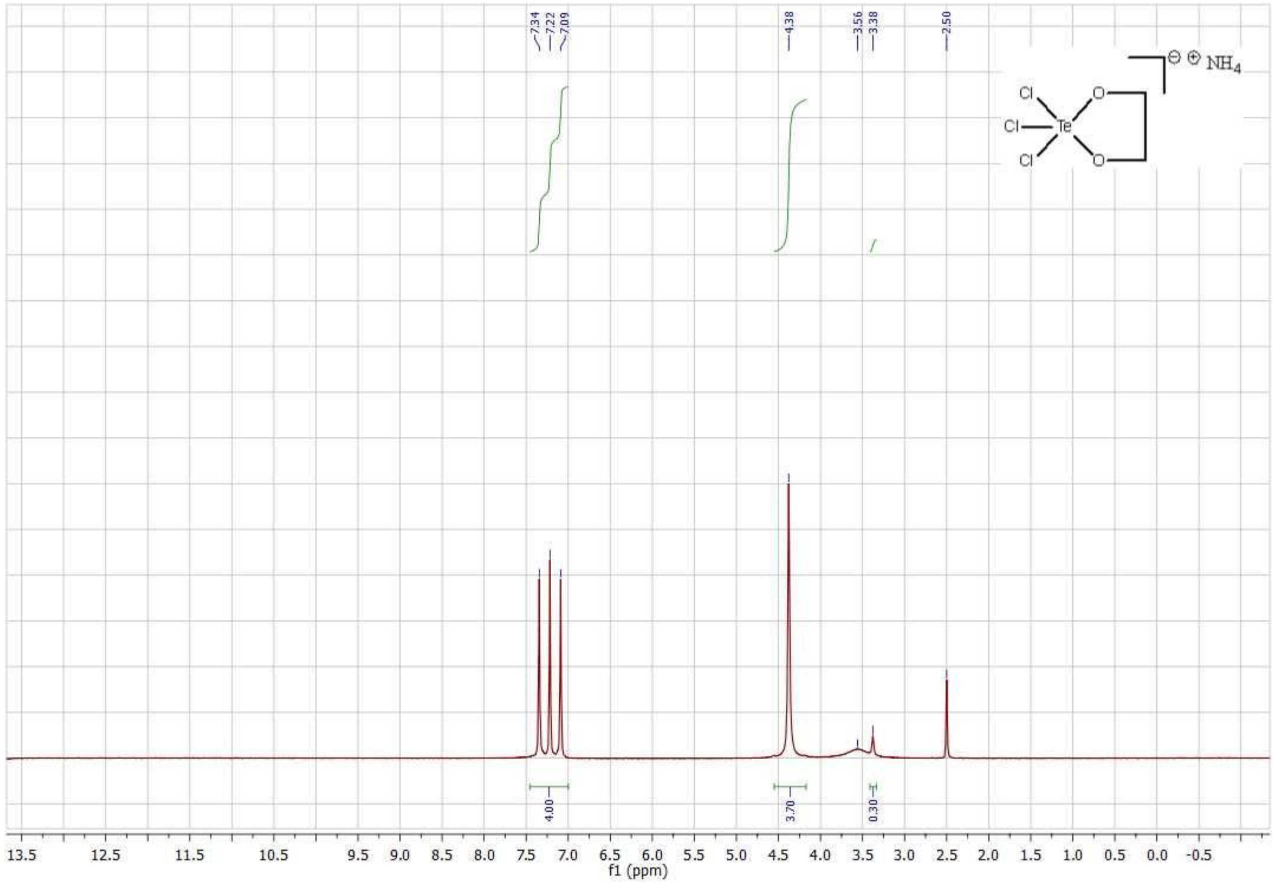

Figure S1. ^{1}HNMR (400 MHz; DMSO-d_6) δ: 7.22 (4H; NH_4^+); 4.38 (s; 4H; CH_2, *coordinated ethylene glycol*); 3.56 (br; H_2O); 3.38 (s, CH_2, *free ethylene glycol, due to partial degradation of complex owing to water in the organic solvent*).

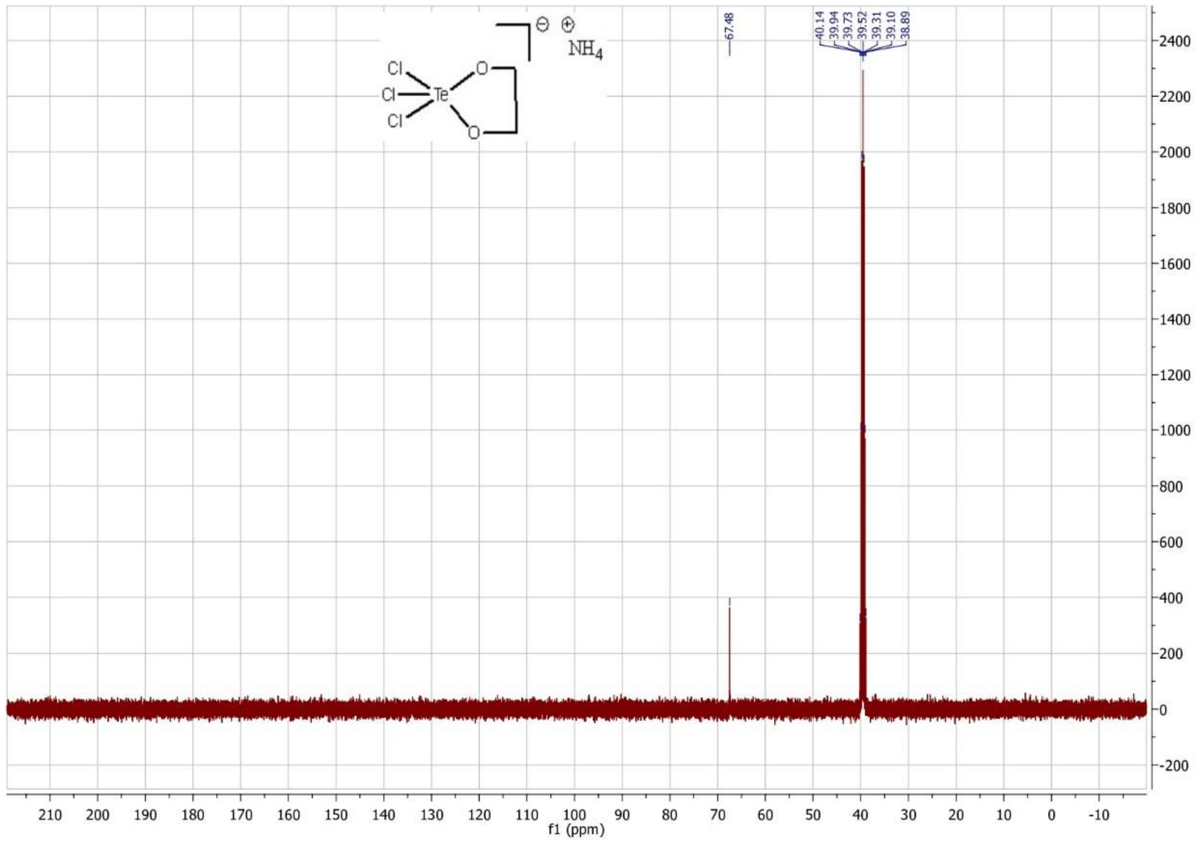

Figure S2. $^{13}C\{^{1}H\}$NMR (100 MHz; DMSO-d_6) δ: 67.48 (CH_2)

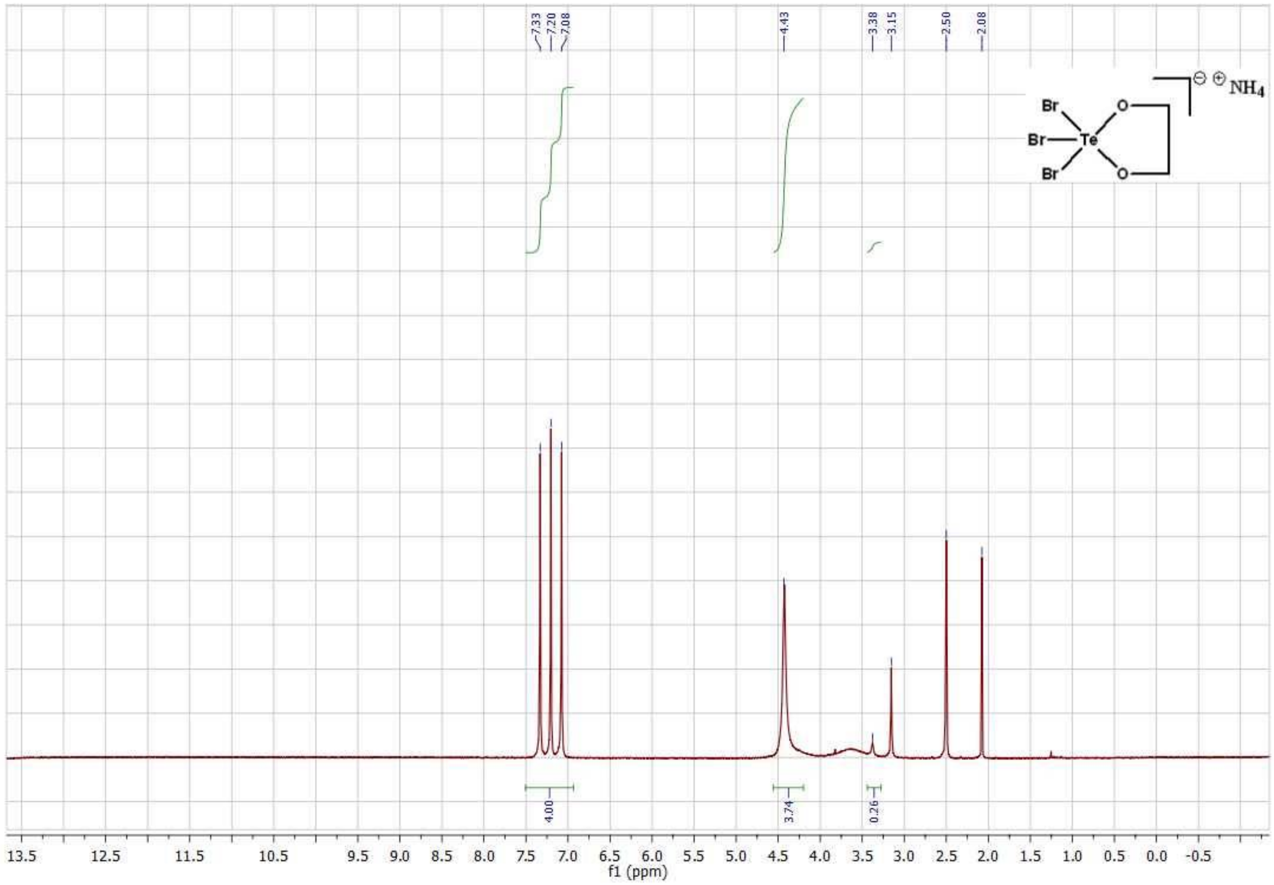

Figure S3. ^{1}HNMR (400 MHz; DMSO-d_6) δ: 7.20 (4H; NH_4^+); 4.43 (s; 4H; CH_2, *coordinated ethylene glycol*). 3.63 (br, H_2O); 3.37 (s, CH_2; *free ethylene glycol, due to partial degradation of complex owing to water in the organic solvent*); 3.15 (methanol; reaction solvent residual); 2.08 (acetone, residual peak of solvent).

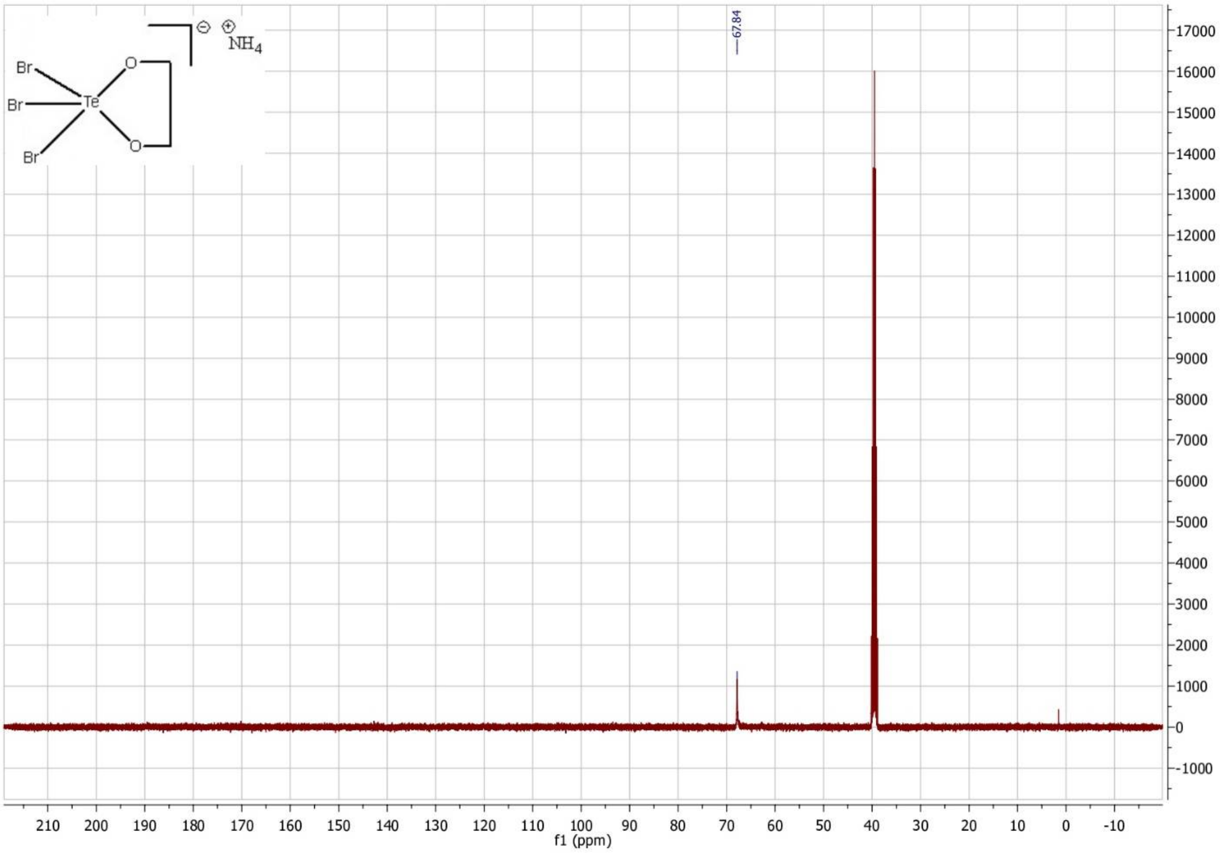

Figure S4. $^{13}C\{^{1}H\}$NMR (100 MHz; DMSO-d_6) δ: 67.84 (CH_2).

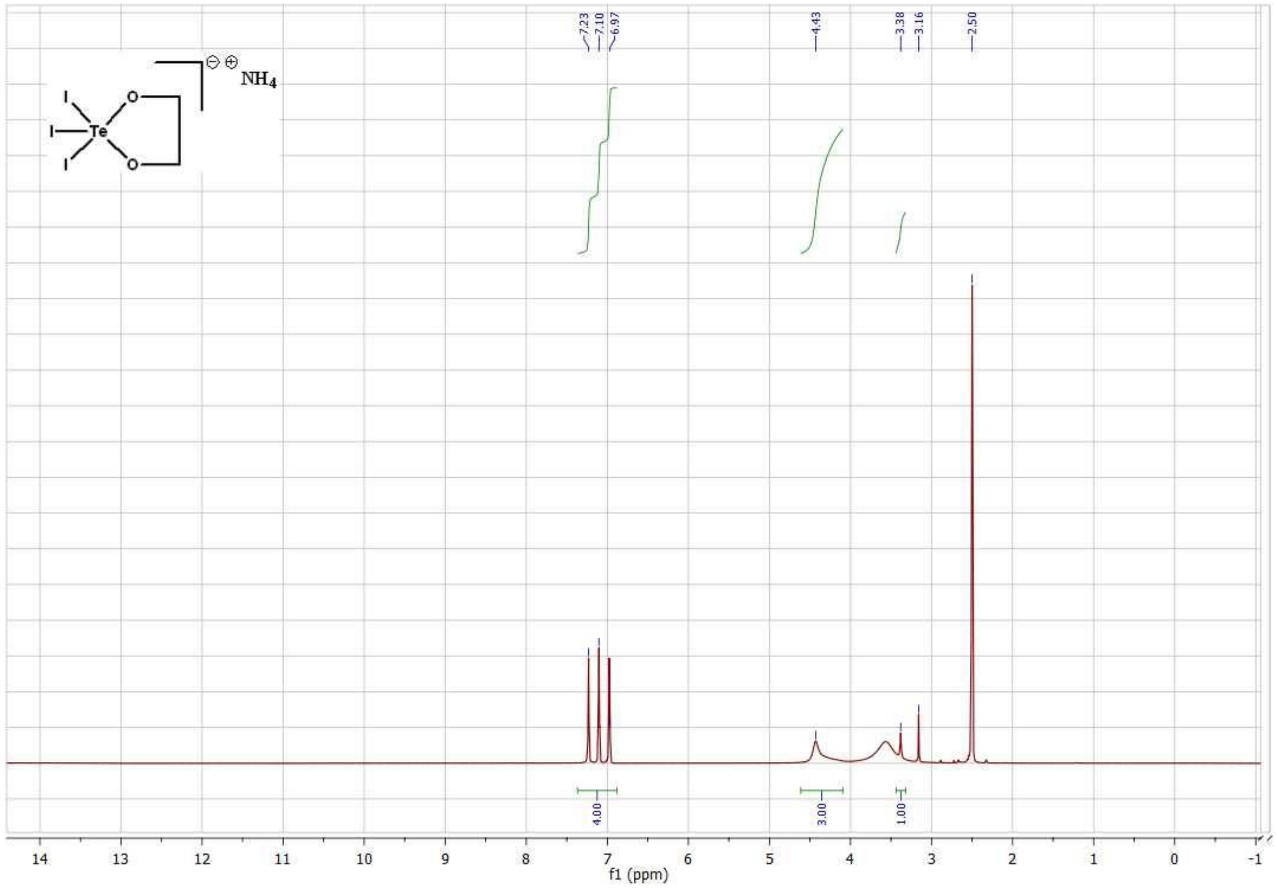

Figure S5. ^{1}HNMR (400 MHz; DMSO-d_6) δ: 7.10 (4H; NH_4^+); 4.43 (s; 4H; CH_2, *coordinated ethylene glycol*). 3.59 (br, H_2O); 3.38 (s; CH_2; *free ethylene glycol, due to partial degradation of complex owing to water in the organic solvent*); 3.16 (methanol; reaction solvent residual).

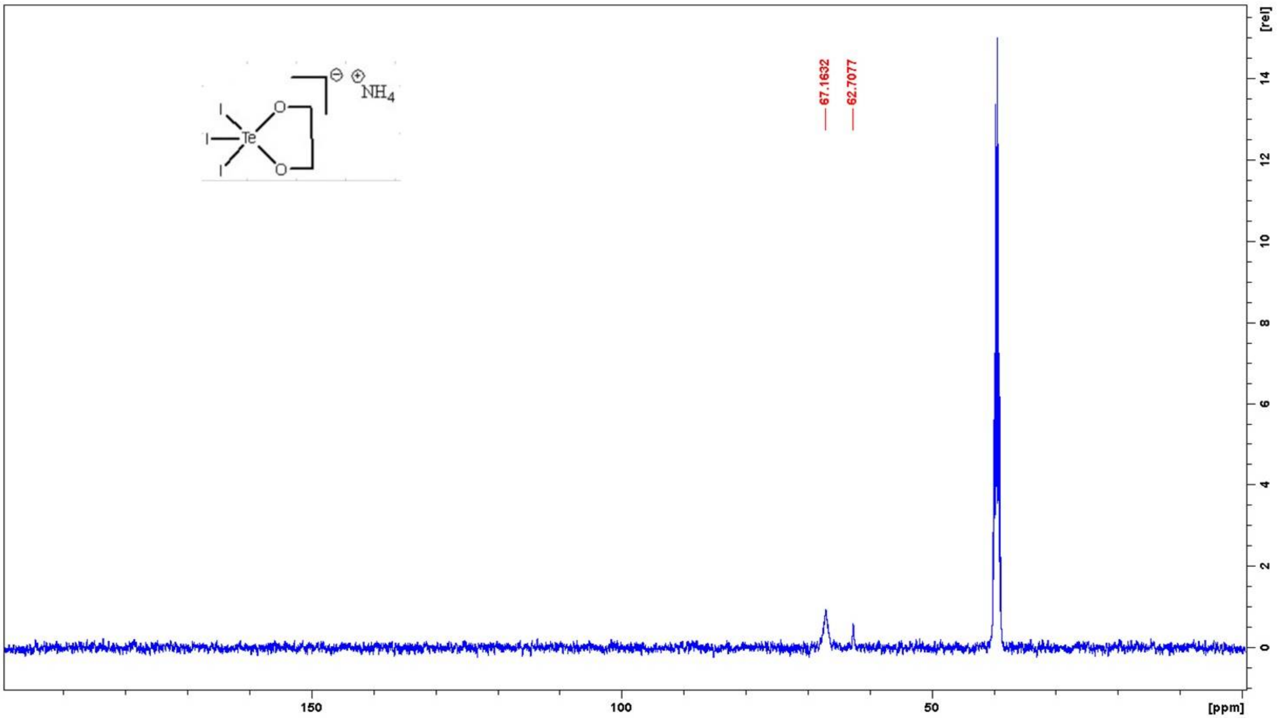

Figure S6. $^{13}C\{^{1}H\}$NMR (100 MHz; DMSO-d_6) δ: 67.16 (CH_2). The signal at 62.70 is attributable to the free ethylene glycol due to partial degradation of complex owing to water in the organic solvent.

IR spectra

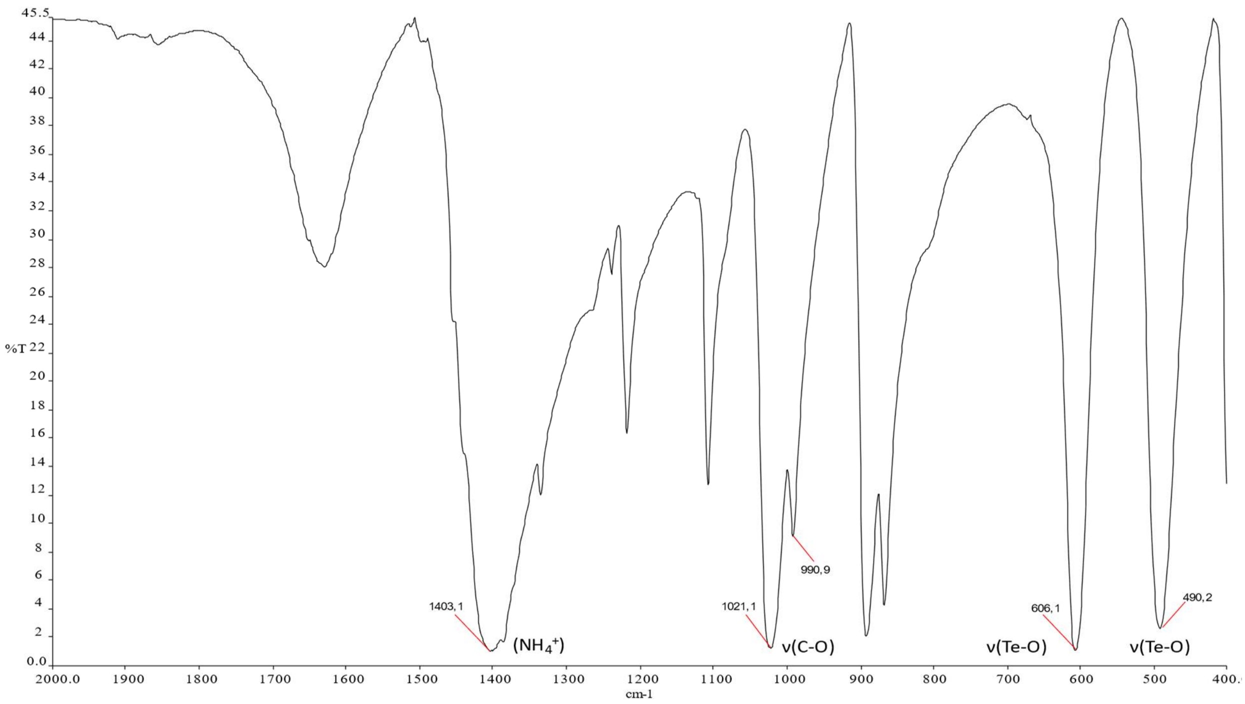

Figure S7. FT-IR spectrum of Ammonium Trichloro(dioxoethylene-O,O')Tellurate (AS101).

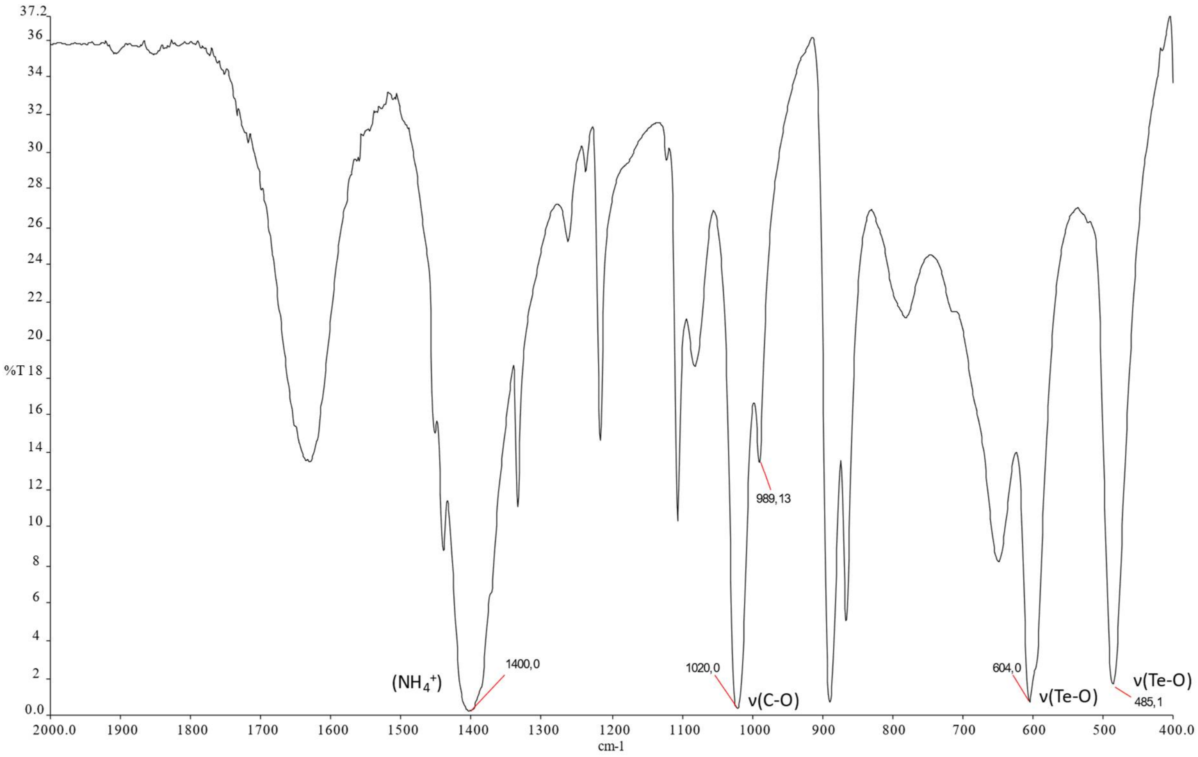

Figure S8. FT-IR spectrum of Ammonium Tribromo(dioxoethylene-O,O')Tellurate.

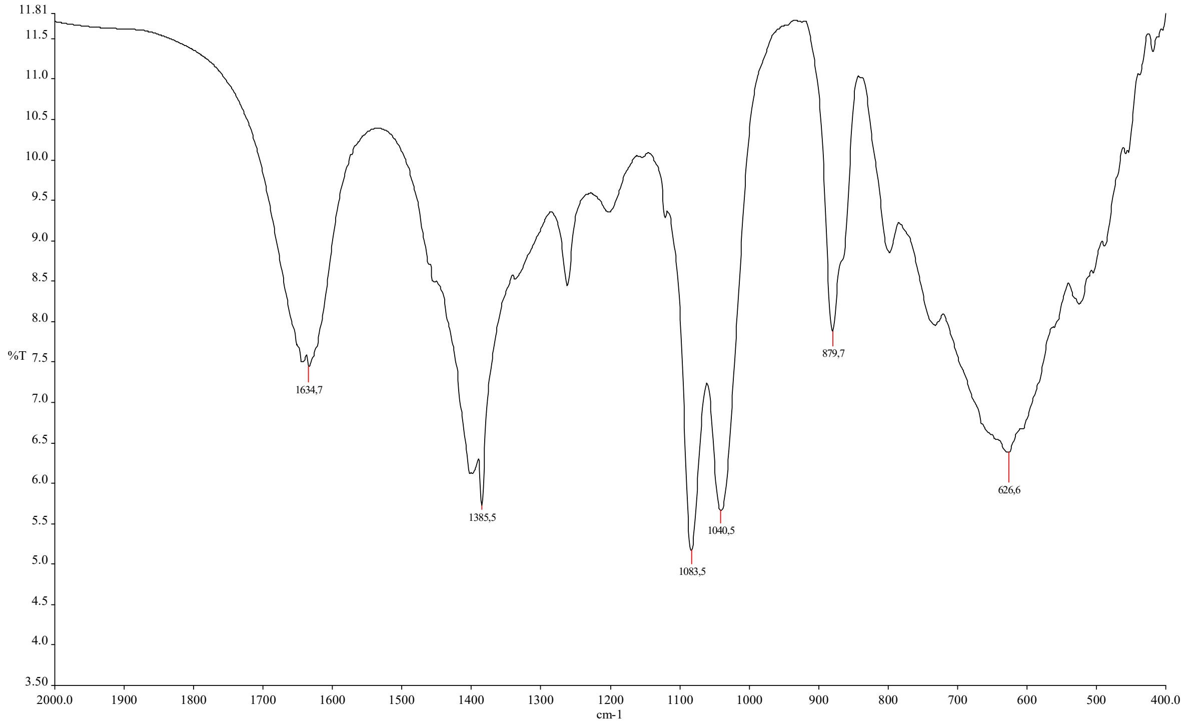

Figure S9. FT-IR spectrum of Ammonium Triiodo(dioxoethylene-O,O')Tellurate. The compound hydrolyzes on KBr tablet to the respective activate form (NH_4TeOI_3).

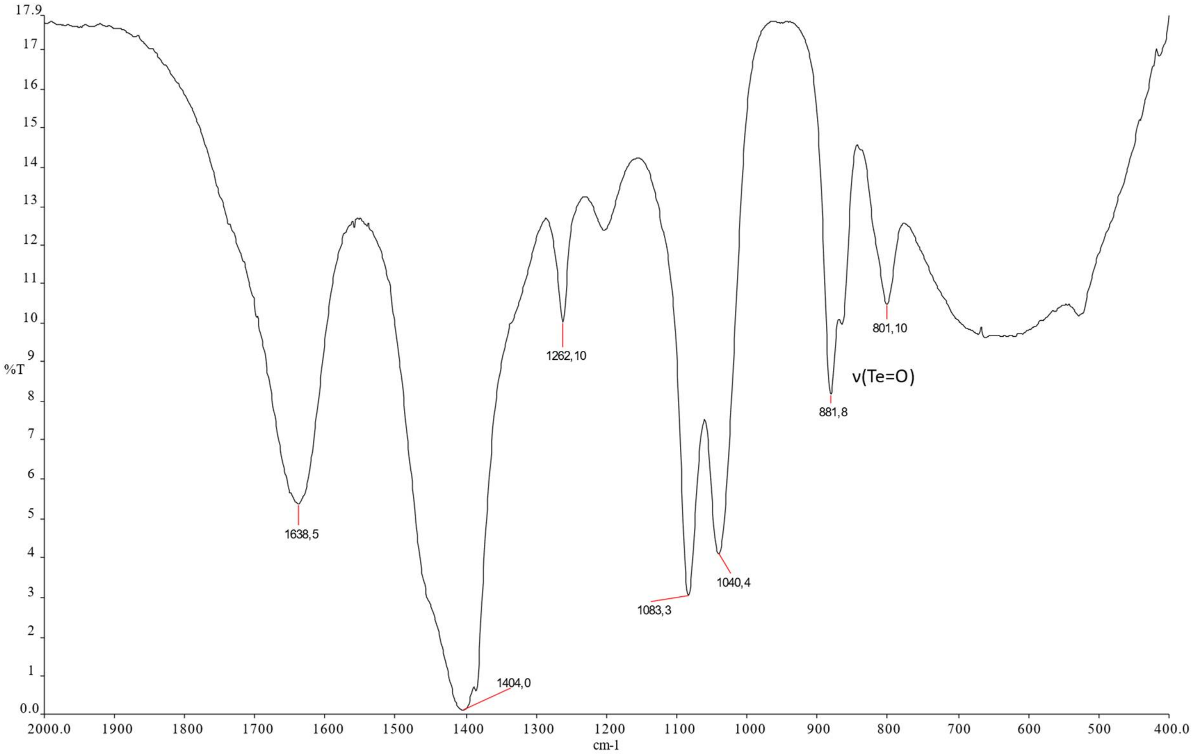

Figure S10. FT-IR spectrum of NH_4TeOCl_3.

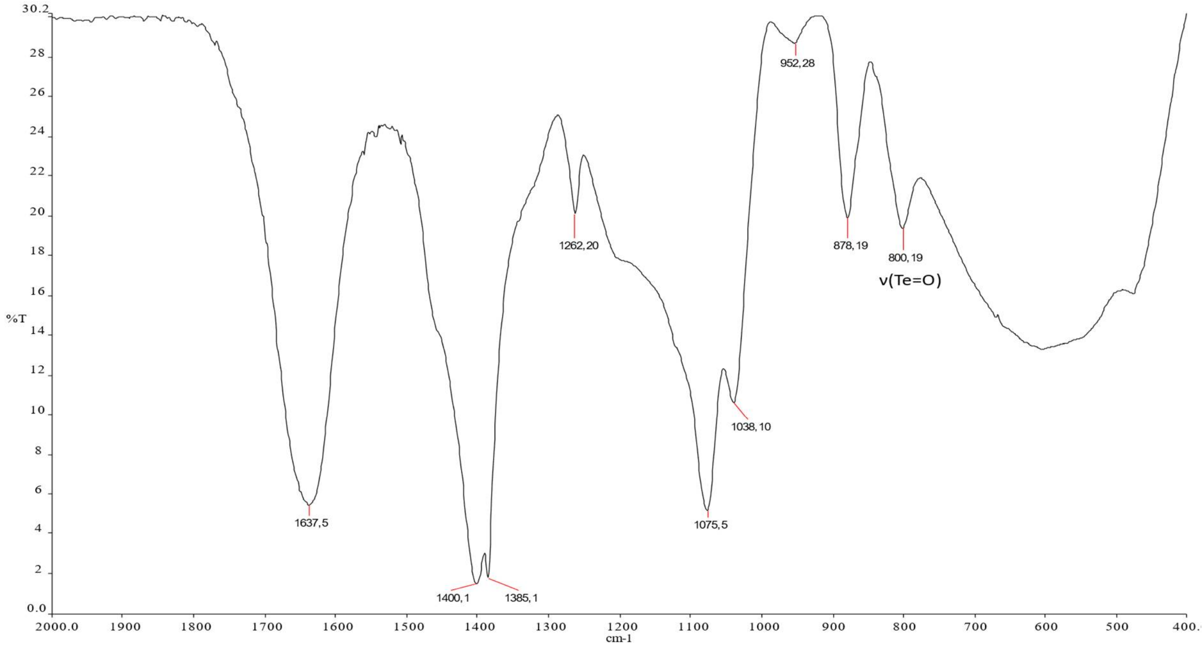

Figure S11. FT-IR spectrum of NH_4TeOBr_3.

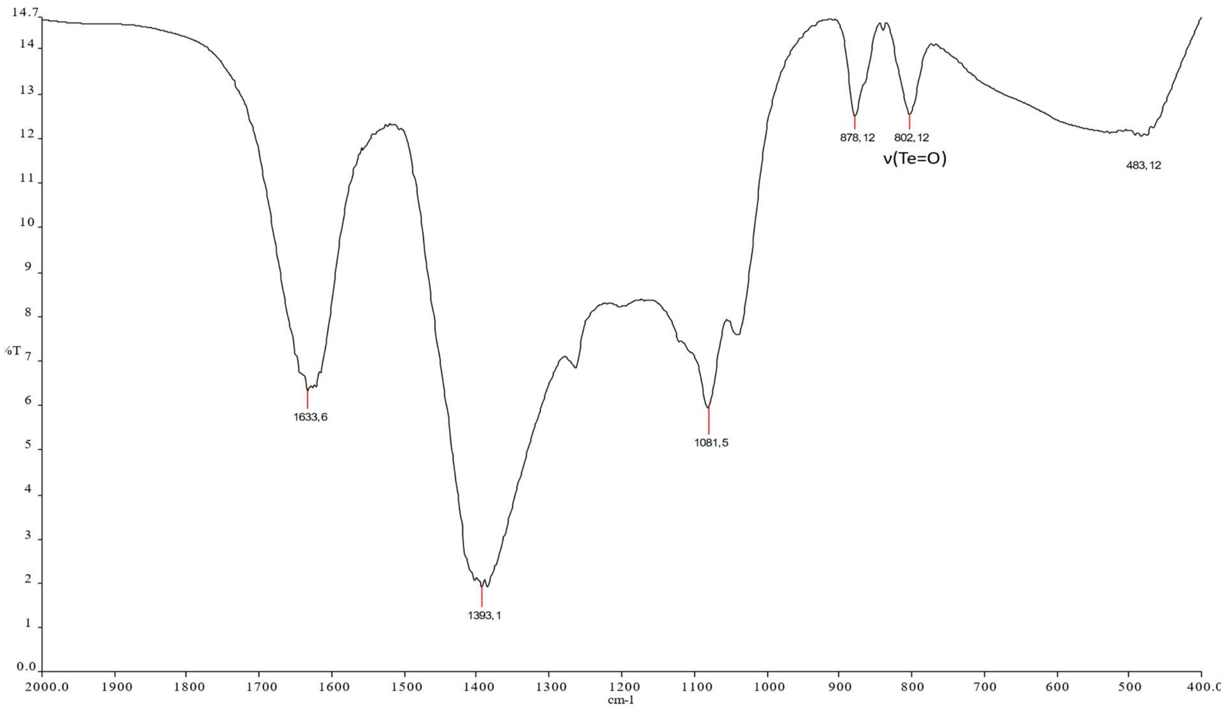

Figure S12. FT-IR spectrum of NH_4TeOI_3

Isolation of the oxidized species

Before IR analysis, the oxidized species were obtained using the same procedure.

To a small quantity (ca. 60 mg) of the selected tellurium compound, 4 mL of deionized water were added. The mixture was magnetically stirred for 2 h at room temperature and then filtered. The resulting solution was evaporated under reduced pressure and the solid obtained dried under vacuum for several hours.

Computational studies

The molar excess of water with respect to the substrate was also taken into account as follows. Let us assume the second-order rate equation:

$$v = k[\mathrm{H_2O}][substrate]$$

By assuming a water excess of:

$$[\mathrm{H_2O}] = 20 \times [substrate]$$

The equation rate can be rewritten:

$$v = 20k[substrate]^2 = k'[substrate]^2$$

Thus, being the *k'* constant corresponding to *20k*, the related activation barrier, i.e. corrected by the water excess, can be calculated by applying the Eyring equation for the uncorrected (i.e. 1:1 water:substrate molar ratio):

$$\ln(k) = -\frac{\Delta G(uncorrected)^{\mp}}{RT} + \ln\left(\frac{k_B T}{h}\right)$$

And 20:1 water substrate molar ratio:

$$\ln(20k) = -\frac{\Delta G(corrected)^{\mp}}{RT} + \ln\left(\frac{k_B T}{h}\right)$$

$$\ln(20) + \ln(k) = -\frac{\Delta G(corrected)^{\mp}}{RT} + \ln(\frac{k_B T}{h})$$

By comparing the above equations, one obtains:

$$\ln(20) + -\frac{\Delta G(uncorrected)^{\mp}}{RT} + \ln\left(\frac{k_B T}{h}\right) = -\frac{\Delta G(corrected)^{\mp}}{RT} + \ln(\frac{k_B T}{h})$$

And by simplifying the last expression, it is finally obtained:

$$\Delta G(corrected)^{\mp} = \Delta G(uncorrected)^{\mp} - RT\ln(20)$$

Table S1. The reaction free energy (kcal/mol) and activation barriers (corrected/uncorrected) for the three-step hydrolysis of AS101 (Cl) and its Br and I derivatives. Format: "computed value corrected for water in excess (20-fold excess) / uncorrected value".

	Barrier for	Cl	Br	I
R->P		13.0	9.9	9.4
R->TS	attack of 1st water	20.5/22.2	16.0/17.7	15.6/17.3
	attack of 2nd water	19.7/21.4	12.9/14.6	12.2/13.9
	detachment of water	17.0/18.7	12.0/13.7	12.7/14.4

Biological studies

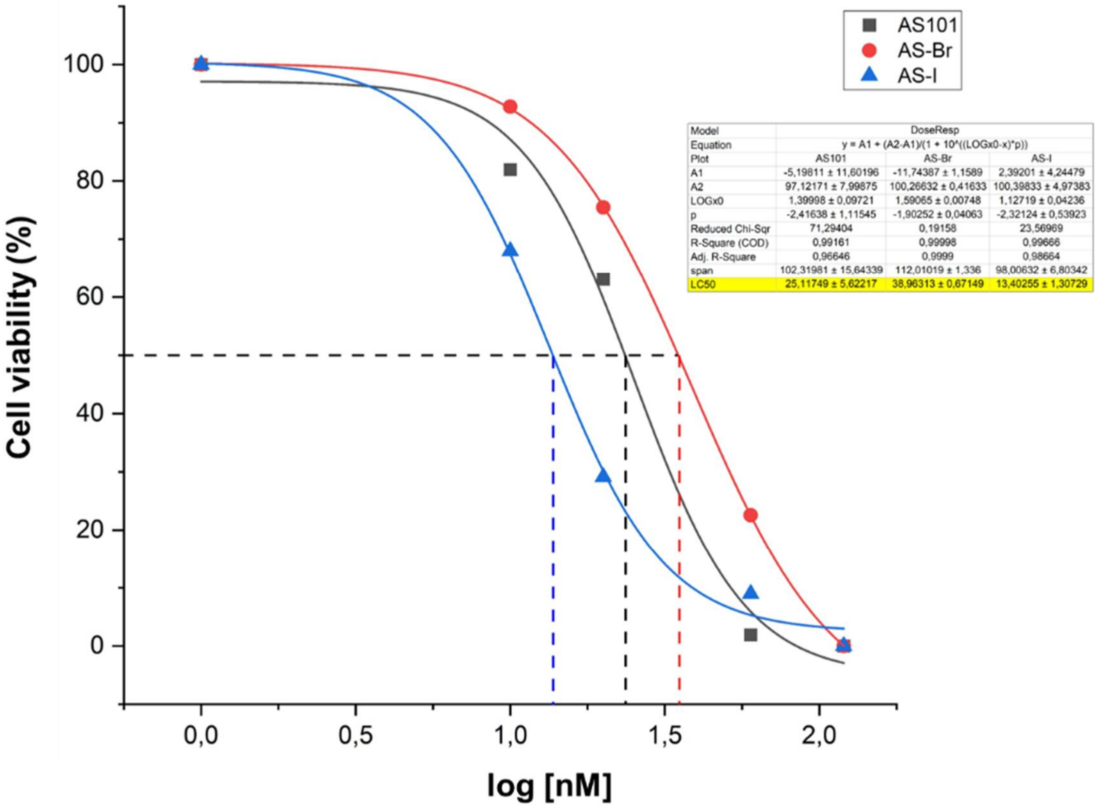

LC_{50} determination. LC_{50} was analyzed by CellTiter 96 Aqueous – One solution Reagent. Cells were treated for 24h with compound at various concentration (1-10-20-60-120nM). Values in the graph indicate the Cell Viability as the mean ± SE obtained from a n=3 of independent experiments; Statistics: One-way Anova test followed by Levene’s post-test.
